# Abdominal injuries in a low trauma volume hospital - a descriptive study from northern Sweden

**DOI:** 10.1186/s13049-014-0048-0

**Published:** 2014-08-15

**Authors:** Patrik Pekkari, Per-Olof Bylund, Hans Lindgren, Mikael Öman

**Affiliations:** 1 Department of Anaesthesiology and Intensive Care, Sunderby Hospital, Lulea, SE-971 80, Sweden; 2 Department of Surgical and Perioperative Sciences; Surgery, Umea University, Umea, SE-901 85, Sweden; 3 Department of Radiology, Umea University, Umea, SE-901 85, Sweden

**Keywords:** Abdominal injuries, Low trauma volume hospital, Non-operative management

## Abstract

**Background:**

Abdominal injuries occur relatively infrequently during trauma, and they rarely require surgical intervention. In this era of non-operative management of abdominal injuries, surgeons are seldom exposed to these patients. Consequently, surgeons may misinterpret the mechanism of injury, underestimate symptoms and radiologic findings, and delay definite treatment. Here, we determined the incidence, diagnosis, and treatment of traumatic abdominal injuries at our hospital to provide a basis for identifying potential hazards in non-operative management of patients with these injuries in a low trauma volume hospital.

**Methods:**

This retrospective study included prehospital and in-hospital assessments of 110 patients that received 147 abdominal injuries from an isolated abdominal trauma (n = 70 patients) or during multiple trauma (n = 40 patients). Patients were primarily treated at the University Hospital of Umeå from January 2000 to December 2009.

**Results:**

The median New Injury Severity Score was 9 (range: 1–57) for 147 abdominal injuries. Most patients (94%) received computed tomography (CT), but only 38% of patients with multiple trauma were diagnosed with CT < 60 min after emergency room arrival. Penetrating trauma caused injuries in seven patients. Solid organ injuries constituted 78% of abdominal injuries. Non-operative management succeeded in 82 patients. Surgery was performed for 28 patients, either immediately (n = 17) as result of operative management or later (n = 11), due to non-operative management failure; the latter mainly occurred with hollow viscus injuries. Patients with multiple abdominal injuries, whether associated with multiple trauma or an isolated abdominal trauma, had significantly more non-operative failures than patients with a single abdominal injury. One death occurred within 30 days.

**Conclusions:**

Non-operative management of patients with abdominal injuries, except for hollow viscus injuries, was highly successful in our low trauma volume hospital, even though surgeons receive low exposure to these patients. However, a growing proportion of surgeons lack experience in decision-making and performing trauma laparotomies. Quality assurance programmes must be emphasized to ensure future competence and quality of trauma care at low trauma volume hospitals.

## Background

Patients with severe abdominal injuries after a blunt or penetrating trauma appear infrequently in most hospitals in Sweden. During the last few decades, management of blunt abdominal injuries has changed from an aggressive surgical approach to non-operative management (NOM), because high quality computerized tomography scanning (CT) provides the ability to readily recognize and follow abdominal injuries [1],[2]. Furthermore, advances in catheter-based technologies have expanded the indications for interventional radiology and facilitated transcutaneous angiographic embolization (TAE). TAE is a widely accepted adjunct in managing solid organ injuries with on-going intra-abdominal or retroperitoneal haemorrhage [3],[4]. However, as a result of these trends in abdominal trauma management, a growing proportion of surgeons at low trauma volume hospitals do not gain sufficient experience in performing trauma laparotomies. Due to the scarcity of these injuries and the increasing lack of experience in open surgical procedures in low trauma volume hospitals, there is a need to enhance the local dataset with defined epidemiology, management strategies, and patient outcomes; this enhancement will facilitate identification of potential hazards in NOM. The aim of this study was to evaluate the incidence, the mechanism of injury, the prehospital time period, the diagnostic workup, and the application of adequate treatment for abdominal injuries, to provide a basis for identifying potential hazards in non-operative management of traumatic abdominal injuries at our low trauma volume hospital.

## Methods

The University Hospital of Umeå is a tertiary referral centre for all of northern Sweden. It serves a population of 880 000 inhabitants in the tertiary catchment area of 225 000 km^2^. This catchment area corresponds to the area of the United Kingdom, with transferral distances from 110 up to 600 kilometres. Because the hospital is the only referral centre in the primary catchment area, which has a population of 145 000 within an 80 km radius, 100% of patients with higher grade trauma are referred.

A computerized trauma registry (Injury Database) of patients primarily treated at the University Hospital of Umeå, has been prospectively maintained since 1985. For the present study, we retrospectively collected data from the Injury Database regarding all patients with a hospital admission from January 2000 to December 2009 for abdominal injuries, with or without injuries in other body regions. Written informed consent was obtained from all patients. The study was a clinical quality-control study approved by the Head of the Department of Surgery, Umeå University Hospital, Umeå, Sweden. The study follows the guidelines of the revised UN declaration of Helsinki in 1975 and its latest amendment in 2008 (6th revision). Permission to use the Injury Database was obtained from the County Council’s Research Injury Database Committee. The study was performed at the Umeå University Medical School, Umeå, Sweden. Patients without abdominal injuries from the primary catchment area, or referred patients with or without abdominal injuries primarily resuscitated at the local hospital in the secondary or tertiary catchment area, were not included in the study. These two groups of patients with AIS3+ injuries constituted about 230 patients annually during the audit period.

Time logs from the prehospital response, emergency unit, department of radiology, and surgery were used to evaluate the efficiency of prehospital and in-hospital trauma care. Patient characteristics included age, gender, mechanism of injury, diagnostic methods, radiologic examination, surgical interventions, and length of hospital stay, including time spent in the intensive care unit (ICU).

Injury severity was classified according to the Injury Scaling and Scoring System [5] and the Abbreviated Injury Scale (AIS) 2005 [6], which appraises the risk of death. The AIS range is 1–6, where AIS1 = minor, AIS2 = moderate, AIS3 = serious, and AIS4-6 = severe, critical, and maximal injuries. AIS grading was completed and coded by licensed AIS specialists. The New Injury Severity Score (NISS) calculates the sum of squares of the top three AIS scores, regardless of body region, providing a score of 0 – 75. Patients with NISS > 15 are classified as seriously injured. NISS is regarded appropriate for patients with multiple injuries within the same anatomic region [7], which describes the patients in our study. A patient with multiple trauma was defined as having injuries in two or more different anatomic sites; a patient with an isolated abdominal trauma had injuries confined to the abdominal cavity or retroperitoneal space.

Descriptive data are expressed in terms of the number (percent) or median (range). Calculations were performed with SPSS 21.0.0.0 (SPSS Inc. Chicago, IL, USA) and Minitab® 16.1.0 (Informer Technologies Inc., http://minitab.software.informer.com). Fisher’s exact test was used to compare proportions between groups, and a p-value less than 0.05 was considered statistically significant.

## Results

Over the ten year period of 2000–2009, we identified 110 patients (n = 75 men) with traumatic abdominal injuries that were primarily treated as in-patients at our hospital. The median age was 21 (6–88) years; 42 patients were <18 years old, 58 patients were 18–65 years old, and 10 patients were >65 years old. Seventy patients experienced isolated abdominal trauma and 40 patients experienced multiple trauma; 87 patients had a single abdominal injury (SAI) and 23 patients had multiple abdominal injuries (MAI). There were significantly more SAI in patients with isolated abdominal trauma than in those with multiple trauma (p < 0.05), and patients with MAI had NISS > 15 more frequently than NISS < 15 (p < 0.05). The mechanism of injury was blunt trauma in 103 patients (94%), and penetrating trauma in seven patients (six stab wounds and one gunshot wound). Nearly 50% of patients were injured in vehicle-related crashes (Table 1).

**Table 1:** Characteristics of 110 patients with multiple trauma or isolated abdominal trauma

	**Multiple trauma**		**Isolated abdominal trauma**		**All trauma**		**Patients**
**Patients**	**MAI**	**SAI**	**MAI**	**SAI**	**MAI**	**SAI**	**Total**
Male (n)	10	15	6	44	16	59	**75**
Female (n)	5	10	2	18	7	28	**35**
Male age in year (range)	35 (16–71)	30 (8–71)	19 (12–48)	21 (8–81)	27 (12–71)	21 (8–81)	**22 (8–81)**
Female age in year (range)	48 (12–72)	16 (11–88)	49 (36–62)	14 (6–84)	48 (12–72)	14 (6–88)	**15 (6–88)**
**Mechanism (n)**	**MAI**	**SAI**	**MAI**	**SAI**	**MAI**	**SAI**	**Total**
Blunt	13*	25*	6*	59*	19	84	**103**
Penetrating	2	0	2	3	4	3	**7**
**Severity**	**MAI**	**SAI**	**MAI**	**SAI**	**MAI**	**SAI**	**Total**
NISS > 15 (n)	12	11	5	13	17*	24*	**41**
NISS < 15 (n)	3	14	3	49	6*	63*	**69**
NISS (range)	27 (12–57)	14 (1–43)	20 (8–36)	4 (1–18)	27 (8–57)	18 (1–43)	**9 (1–57)**
**Circumstance (n)**	**MAI**	**SAI**	**MAI**	**SAI**	**MAI**	**SAI**	**Total**
Vehicle related	7	18	4	25	11	43	**54**
Fall	5	6	1	19	6	25	**31**
Assault	2	0	1	6	3	6	**9**
Other	1	1	2	12	3	13	**16**

Legends: n = number of patients. MAI = Multiple abdominal injury. SAI = Single abdominal injury. Age in median (range). NISS = New Injury Severity Score. NISS in median (range). * = p < 0.05.

### Prehospital emergency care

Prehospital response was provided for 56 patients; the response team arrived at the scene in < 15 min (median: 12 min; range: 5–49) for 34 (61%) patients. Prehospital response was significantly more often activated for patients with multiple trauma compared to those with isolated abdominal trauma (p < 0.05). The time spent at the scene was < 15 min (median: 13 min; range: 5–36) for 36 patients (64%). The time from the alert to the arrival at the emergency room (ER), was < 60 min (median: 45 min; range: 8–141) for 42 patients (75%) (Table 2). Of the 33 patients injured in vehicle crashes, 15 received prehospital care at the scene for > 15 min. Among the 56 patients with a prehospital response, 55% had NISS scores >15; in contrast 19% of patients that arrived to the hospital by private car or taxi (n = 54) had NISS scores >15.

**Table 2:** Prehospital response, time and treatment in 110 patients with multiple trauma or isolated abdominal trauma

	**Multiple trauma**		**Isolated abdominal trauma**		**All trauma**		**Patients**
**Response**	**MAI**	**SAI**	**MAI**	**SAI**	**MAI**	**SAI**	**TOTAL**
PR No	1*	6*	2*	45*	3	51	**54**
PR Yes	14*	19*	6*	17*	20	36	**56**
CT No	2	1	2	2	4	3	**7**
CT Yes	13	24	6	60	19	84	**103**
RT <15	9	10	3	12	12	22	**34**
TOS <15	8	12	3	13	11	25	**36**
TER <60	12	13	5	12	17	25	**42**
TCT <60	7	8	1	9	8	17	**25**
**Time**	**MAI**	**SAI**	**MAI**	**SAI**	**MAI**	**SAI**	**TOTAL**
RT	10 (5–49)	13 (5–37)	14 (5–16)	10 (5–40)	12 (5–49)	12 (5–40)	**12 (5–49)**
TOS	14 (5–34)	13 (5–36)	15 (5–20)	7 (5–20)	14 (5–34)	11 (5–36)	**13 (5–36)**
TER	42 (17–141)	48 (12–85)	42 (15–69)	38 (8–100)	42 (15–141)	45 (8–100)	**45 (8–141)**
TCT	43* (28–110)	71* (9–1277)	92* (57–1035)	115* (17–1153)	74 (28–1035)	98 (9–1277)	**94 (9–1277)**
**Patients**	**MAI**	**SAI**	**MAI**	**SAI**	**MAI**	**SAI**	**TOTAL**
OM	5	4	3	5	8	9	**17**
NOM-S	7	19	3	53	10*	72*	**82**
NOM-F	3	2	2	4	5*	6*	**11**
**Total**	**15**	**25**	**8**	**62**	**23**	**87**	**110**

Legends: MAI = Multiple abdominal injury. SAI = Single abdominal injury. PR = Prehospital Response. Time in minutes. < 15 = less than 15 minutes. < 60 = less than minutes. TOS = Time on scene. CT = Computerized tomography. RT = Response time denotes time from alert to arrival at scene. TER = Time to ER denotes time from alert to arrival at the emergency room, i.e. total prehospital time. OM = Operative Management. NOM-S = Non Operative Management Success. NOM-F = Non Operative Management Failure. TCT = Time to CT denotes time from arrival at ER to start of CT examination. Time in median (range). * = p < 0.05.

### Diagnostics

A total of 103 patients (94%) with multiple or isolated abdominal trauma were initially diagnosed with a CT after 67 (range: 9–1277) and 114 (range: 17–1153) min in the ER, respectively. Of 37 patients with multiple trauma and 66 patients with isolated abdominal trauma, 15 (41%) and 10 (15%), respectively, were examined with CT within 60 min from arrival at the ER. The time from ER arrival to the start of a CT examination was significantly shorter among patients with multiple trauma than among those with isolated abdominal trauma (p < 0.05) (Table 2). Seven patients were judged to require immediate surgery and went directly to the operating room (OR) without radiologic examination, except for an anterior-posterior chest and pelvis X-ray in the ER. These patients either had penetrating injuries and/or showed signs of abdominal injury with circulatory instability, despite fluid resuscitation. Two patients in this group underwent a positive diagnostic peritoneal lavage in the ER; one patient with a penetrating perineal injury, but stable circulation, underwent a sigmoidoscopy prior to surgery.

### Injuries and treatment

Among all 110 patients, 147 abdominal organ AIS1+ injuries were found. In addition, there were 109 extra-abdominal injuries in the multiple trauma group, which resulted in a total median NISS of 9 (range: 1–57). In patients with multiple and isolated abdominal trauma, 23 (58%) and 40 (57%) had abdominal AIS3+ injuries, respectively; thus, the NISS was >15 in 58% and 26%, respectively. In patients with multiple and isolated abdominal trauma, MAI were found in 38% and 11%, respectively. Patients with isolated abdominal trauma had substantially more SAI than MAI (Table 1).

Seventeen patients received primary operative management. NOM was initiated in 93 patients (85%), but failed in eleven patients. Among the latter, six had hollow viscus injuries, four had on-going haemorrhages from solid organ injuries, and one had a pancreatic duct disruption (Table 3).

**Table 3:** Operative and non-operative management of 110 patients with 147 abdominal injuries in AIS grade

**AIS grade**	**NOM**	**NOM-F**	**OM**	**Total**
	**≤2/≥3**	**≤2/≥3**	**≤2/≥3**	**≤2/≥3**
**Solid organ injury (n = 115)**				
Kidney (n = 39)	22/17	0	0	**22/17**
Liver (n = 34)	21/10	0/1	0/2	**21/13**
Spleen (n = 31)	15/11	1/2	0/2	**16/15**
Adrenal gland (n = 9)	8/1	0	0	**8/1**
Pancreas (n = 2)	1/0	0/1	0/0	**1/1**
**Hollow viscus injury (n = 17)**				
Small intestine (n = 9)	0	1/3	1/4	**2/7**
Colon (n = 5)	0	1/1	1/2	**2/3**
Bladder (n = 2)	0	0	0/2	**0/2**
Rectum (n = 1)	0	0	0/1	**0/1**
**Other injury (n = 15)**				
Abdominal wall (n = 7)	3/0	0	4/0	**7/0**
Diaphragm (n = 1)	0	0	0/1	**0/1**
Abdominal vessel (n = 7)	0	0	2/5	**2/5**
**All injuries (n = 147)**				
Total	**70/39**	**3/8**	**8/19**	**81/66**

Legends: Time to surgery denotes time in hours from arrival at the emergency room to beginning of surgical procedure. AIS = Abbreviated Injury Scale.

There was no difference in the numbers of successful and failed NOM between patients injured in multiple or isolated abdominal trauma, but there were significantly more failures than successes in patients with MAI compared to those with SAI (p < 0.05) (Table 2).

### Solid organ injuries

Solid organ injuries comprised 78% of all injuries. The organs most frequently injured were the kidneys (n = 39), the liver (n = 34), the spleen (n = 31), the small intestine (n = 9), and the mesenteric vessels (n = 7). The NOM was 95% successful among patients with solid organ injuries. NOM was even initiated in 15 patients with solid organ AIS4+ injuries (7 kidney, 5 liver, and 3 spleen injuries); of these, two failed (1 spleen and 1 liver injury) (Table 3).

Spleen injuries were found in 31 patients. Splenectomy was performed in two patients (AIS3 and AIS5 injuries) as part of damage control surgery, and it was performed in three patients (AIS2, AIS3, and AIS4 injuries) due to NOM failure. Twenty-six patients with spleen injuries had successful NOM, but one of these, with an AIS3 injury and on-going haemorrhage, underwent an adjunct TAE.

Liver injuries were found in 34 patients. Open packing of the liver as part of damage control surgery was performed in two patients (AIS4 and AIS5 injuries); of which one had a successful postoperative TAE. One patient (AIS5 injury) failed NOM. Successful NOM were achieved in 31 patients; of these, one (AIS4 injury) underwent endoscopic retrograde cholangiopancreatography with sphincterotomy at 14 days after the injury, and a trans-sphincteric endoprosthesis was placed, due to bile leakage.

All 39 kidney injuries were successfully treated with NOM. One patient (AIS4 injury) with persistent haemorrhage underwent TAE as an adjunct to NOM.

Of two patients with pancreatic trauma, one patient, with an AIS3 injury, underwent laparotomy and distal pancreatectomy, due to distal transection of the pancreatic duct. The other patient, with an AIS2 pancreatic injury, was treated successfully with NOM.

### Hollow viscus injuries and injuries to mesenteric vessels

A total of 14 patients with single or multiple intestinal injuries and/or mesenteric vascular injuries were treated at the hospital during the study period. Of these, eight patients were subjected to surgery within three hours of their arrival to the ER, due to one or several of the following: penetrating injury (n = 2), circulatory instability with signs of abdominal injury (n = 5), peritonitis (n = 3), and/or free intra-peritoneal air detected on radiologic examination (n = 2). Six patients that received an initial CT with no abnormal findings and no clinical signs or symptoms of abdominal injury, failed NOM. These patients underwent exploratory laparotomy after 7–60 h of observation in the ICU or surgical ward. They were taken to the OR, either after deterioration in their clinical condition with peritonitis (n = 5) and/or haemodynamic instability (n = 2), and/or after a follow-up CT had revealed free intra-peritoneal air (n = 3). The initial CT disclosed intraperitoneal bladder injuries in two patients; these underwent surgery 3 and 6 h after arrival to the ER (Table 4). No patients with injuries to hollow organs or to mesenteric vessels were successfully treated with NOM.

**Table 4:** Surgical procedures in 28 patients with 35 abdominal injuries in time intervals and AIS grade

**Time to surgery (hours)**	**<1**	**1-3**	**4-6**	**7-12**	**13-24**	**25-48**	**>48**	**Total**
**AIS grade**	**≤2/≥3**	**≤2/≥3**	**≤2/≥3**	**≤2/≥3**	**≤2/≥3**	**≤2/≥3**	**≤2/≥3**	**≤2/≥3**
Vascular repair	1/1							**1/1**
Splenectomy		0/2		1/0	0/1		0/1	**1/4**
Liver packing*	0/1		0/1	0/1				**0/3**
Distal pancreatectomy				0/1				**0/1**
Bowel repair/resection	0/3	0/3		0/1	0/2	1/1	1/0	**2/10**
Enterostomy		0/2			1/0	0/1	1/0	**2/3**
Bladder repair		0/1	0/1					**0/2**
Diaphragm repair	0/1							**0/1**
Abdominal wall repair^#^		1/0	1/0		1/0		1/0	**4/0**
**TOTAL**	**7**	**9**	**3**	**4**	**5**	**3**	**4**	**35**

Legends: Time to surgery denotes time in hours from arrival at the emergency room to beginning of surgical procedure. AIS = Abbreviated Injury Scale. *Liver packing as part of damage control surgery. ^#^ Two patients underwent abdominal wall repair without laparotomy.

### Other injuries

One patient underwent a combined abdominal and thoracic surgical procedure, due to a penetrating injury across the diaphragm, that caused both liver and lung injuries. Four patients underwent operative management; three were due to penetrating abdominal wall injuries, without coexisting intra-abdominal injuries; and one was due to an abdominal wall defect after blunt trauma.

### Length of hospital stay

Patients that experienced isolated abdominal trauma (n = 70) stayed in the hospital for 5 days (range: 1–28 days). Of these, 21 were admitted to the ICU for 1 day (range: 1–6 days). Patients that experienced multiple trauma (n = 40) stayed in the hospital for 8 days (range: 1–36 days). Of these, 29 were treated in the ICU for 3 days (range: 1–32 days).

### Mortality

One patient experienced multiple trauma and presented at the ER in profound haemorrhagic shock, due to extensive abdominal and pelvic crush injuries (NISS = 57). This patient died of exsanguination 12 h after arrival, despite immediate surgery. The surgical procedures included subdiaphragmatic clamping of the aorta, suturing several large lacerations of the inferior vena cava, pelvic packing, and external fixation of the pelvis.

## Discussion

Our study confirmed that our hospital had a low incidence of patients with abdominal injuries that required surgical intervention. During the study period, around ten patients annually from the primary catchment area of the University Hospital of Umeå were treated for AIS1+ abdominal injuries. The median age was 21 (6–88) years, and the majority of the patients were men (68%). Most patients had been injured in an isolated abdominal trauma (n = 70), which in most cases (79%), caused a single abdominal injury. Nearly 50% of patients were injured in a vehicle-related trauma, and only seven patients (6%) had a penetrating trauma. We have only found a few studies from Scandinavia that described the local incidence and management of abdominal injuries in the adult and paediatric population [8]-[11]. Those studies showed that the male gender was overrepresented and that penetrating injuries were relatively rare, consistent with our findings. A recent two-year review from the Swedish National Trauma Registry that comprised 7200 patients that experienced trauma (median age 38 years), with or without abdominal injuries, reported that men comprised 66.7% of the injured, that more than 50% were injured in vehicle crashes, and that only 6.4% had penetrating injuries [12]. The median age of patients with abdominal injuries in our study was lower than that of the general population of Swedish patients that experienced trauma. Thus, abdominal injuries appeared to affect younger patients rather than older patients, and they often result from an isolated abdominal trauma.

Our study showed that most patients with NISS > 15 arrived at the ER by ambulance or helicopter, and nearly 20% of these patients with severe injuries arrived by private car or taxi. In comparing our study to national Swedish data [12], the prehospital response time was 12 vs. 13 min, the time on scene of was 13 vs. 18 min, and the time from the alert to arrival at the ER was 45 vs. 52 min, respectively. From our perspective, these times were reasonable, given the vast primary catchment area. Among the patients injured in motor vehicle crashes (n = 24), 50% were treated at the scene longer than 15 min, due to prolonged extrication from vehicles.

Seven of 110 patients with abdominal injuries were judged to require immediate surgical intervention, and they were sent to the OR before receiving a CT. Two of these patients underwent a positive diagnostic peritoneal lavage prior to surgery to determine the presence of haemoperitoneum. The ‘Focused Assessment with Sonography in Trauma’ (FAST) approach was not in clinical practice at our hospital during the study period. The remaining 103 patients had stable circulation in the ER and underwent CT in accordance with Advanced Trauma Life Support Guidelines [13]. Ten additional patients were taken to the OR for abdominal surgery shortly after the CT examination.

We found that, for patients with multiple trauma and isolated abdominal trauma, the times between arrival to the ER and the CT examination were 67 and 114 min, and 41% and 15%, respectively, were examined within 60 min. The national data reported the time from the ER to the CT [12] was 48 min, but that estimate excluded patients with times exceeding 200 min. Despite this difference, our prolonged times may reflect a time-consuming element in the initial trauma management that may potentially be improved at our hospital.

In the last two decades, incorporating NOM into solid organ injury treatments was one of the most notable changes in the care for patients after blunt abdominal trauma. NOM of solitary liver, spleen, and renal injuries is considered the standard of care in all injured adults that are haemodynamically stable, without signs of peritonitis. Numerous studies have demonstrated NOM success rates in adult patients that exceeded 80% in spleen, 90% in liver, and 90% in renal injuries [1],[14]-[21]. NOM has also been successful in paediatric patients [22]. Even multiple trauma patients with more than one solid organ injury can be treated with NOM, provided that the patient is haemodynamically stable and carefully monitored, with no signs of peritonitis [2],[23]. In this study, we found significantly more NOM failures than successes among patients with more than one abdominal injury; this finding suggests that patients with MAI should be followed more cautiously when treated with NOM. We found no difference in NOM success and failure rates between patients that experienced multiple or isolated abdominal trauma. The overall NOM success rate was 89%. The NOM failures comprised six patients with delayed diagnoses of hollow viscus perforation, one patient with pancreatic duct disruption, and four patients with persistent solid organ injury haemorrhage. The NOM success rate was 95% among patients with kidney, liver, and spleen injuries; even those with AIS4+ injuries had a 86% NOM success rate. Only three patients with on-going solid organ injury haemorrhage underwent TAE as an adjunct to NOM or surgery. In future, with increasing indications and accessibility to interventional radiology, more patients will be offered treatment with this minimally-invasive technique.

Pancreatic trauma is a special case, because it is a rare injury, and diagnosis requires a high degree of suspicion. NOM in pancreatic trauma remains limited to injuries without ductal disruption, which requires surgery or advanced endoscopy [1],[24],[25]. We found only two pancreatic injuries, and both initially received NOM. NOM failed for one, and after seven hours of observation, distal pancreatectomy was performed, due to ductal disruption.

Early diagnosis of hollow viscus injury may be difficult, because the symptoms are minute and the initial CT indications are subtle [26],[27]. In this study, the diagnosis and surgical management of intestinal perforation was delayed by 7–60 h in 6 of 14 patients.

Among patients that experienced trauma from the primary catchment area of the University Hospital of Umeå, only 2–3 patients, annually, required abdominal surgery. These findings were consistent with a study from Linköping University Hospital, which serves a local population of 260 000, and it serves 835 000 people as a secondary and tertiary referral centre; they found only five patients with trauma required laparotomy over one audit year [11]. At Ullevaal University Hospital in Oslo, the largest trauma centre in Norway, which serves about 2.5 million people, an average trauma team leader participated in ten trauma laparotomies per year [10]. Surgeons that work at low trauma volume hospitals should be enrolled in educational programmes for open surgical procedures and take part in exchange programmes with centres that have high trauma workloads, to ensure the quality of operative trauma care. Technical surgical training simulations have mainly focused on highly technique-dependent, mini-invasive, endoscopic, laparoscopic, percutaneous, or endovascular procedures. There is a scarcity of studies that evaluate models for open surgical simulation, including synthetic prototypes, animal models, or human cadavers, compared to models with a mini-invasive approach. Two recent reviews [28],[29] emphasized the need for more studies on simulation-based teaching techniques to provide data for evaluating how well these models can replace or enhance the traditional surgical training through apprenticeship in the operating theatre.

### Limitations

The low total number of patients and small subgroups in this study limited the feasibility of further statistical analysis. In this register study, it was not possible to analyse the long-term effects of the timing of operative management or NOM failure.

## Conclusions

Surgeons exposed to few abdominal traumas achieve limited experience in the diagnostics and treatment of these injuries. Despite the low incidence of abdominal trauma at our hospital, NOM was successful in 89% of all injuries and in 95% of solid organ injuries. Surgeons working in a low volume trauma hospital like ours, continue to make the right decisions and determine whether a patient should undergo an operative intervention. In time, a new generation of surgeons that are subspecialized and skilled in mini-invasive procedures, but with limited experience in traditional open surgery, will ultimately be responsible for making these decisions. To ensure local competence in performing trauma laparotomies, quality assurance programmes should be implemented to provide laboratory training courses and to sponsor national and international trauma surgery exchange programmes.

## Abbreviations

AIS: Abbreviated Injury Scale

CT: Computed tomography

ER: Emergency room

ICU: Intensive care unit

MAI: Multiple abdominal injuries

NISS: New Injury Severity Score

NOM: Non-operative management

OR: Operating room

SAI: Single abdominal injury

TAE: Transcutaneous angiographic embolization

## Competing interests

The authors declare that they have no competing interests.

## Authors’ contribution

PP conceived the study, gathered and analysed the data, and drafted the manuscript. POB conceived the study, analysed the data, and drafted the manuscript. HL analysed the radiological data and drafted the manuscript. MÖ conceived the study, analysed the data, supervised the conduct of the study, drafted the manuscript, and takes responsibility for the article as a whole. All authors have read and approved the final manuscript.

## References

[B1] Schroeppel TJ Croce MA Diagnosis and management of blunt abdominal solid organ injury Curr Opin Crit Care 2007 13 399 404 10.1097/MCC.0b013e32825a6a32 17599009

[B2] Yanar H Ertekin C Taviloglu K Kabay B Bakkaloglu H Guloglu R Nonoperative treatment of multiple intra-abdominal solid organ injury after blunt abdominal trauma J Trauma 2008 64 943 948 10.1097/TA.0b013e3180342023 18404060

[B3] Wei B Hemmila MR Arbabi S Taheri PA Wahl WL Angioembolization reduces operative intervention for blunt splenic injury J Trauma 2008 64 1472 1477 10.1097/TA.0b013e318174e8cd 18545111

[B4] Holden A Abdomen–interventions for solid organ injury Injury 2008 39 1275 1289 10.1016/j.injury.2008.04.019 18715559

[B5] http://www.aast.org/library/traumaprevention/injurypreventionguide.aspx AAST: **Injury scaling and scoring system.** 2014, [] Accessed February.

[B6] Genarelli T, Wodzin Z Abbreviated Injury Scale 2005, updated 2008 2008 1 Association for the Advancement of Automotive Medicine, Barrington, IL, USA

[B7] Balogh Z Offner PJ Moore EE Biffl WL NISS predicts postinjury multiple organ failure better than the ISS J Trauma 2000 48 624 627 10.1097/00005373-200004000-00007 10780593

[B8] Sjovall A Hirsch K Blunt abdominal trauma in children: risks of nonoperative treatment J Pediatr Surg 1997 32 1169 1174 10.1016/S0022-3468(97)90676-X 9269964

[B9] Franzen L Ortenwall P Backteman T Major trauma with multiple injuries in Swedish children Eur J Surg Suppl 2003 588 3 7 15200035

[B10] Gaarder C Skaga NO Eken T Pillgram-Larsen J Buanes T Naess PA The impact of patient volume on surgical trauma training in a Scandinavian trauma centre Injury 2005 36 1288 1292 10.1016/j.injury.2005.06.034 16122752

[B11] Al Ayoubi F Eriksson H Myrelid P Wallon C Andersson P Distribution of emergency operations and trauma in a Swedish hospital: need for reorganisation of acute surgical care? Scand J Trauma Resusc Emerg Med 2012 20 66 10.1186/1757-7241-20-66 22985447 PMC3568729

[B12] http://www.swetrau.se/dokument-2 Troëng T, Brattström O: **Swedish trauma registry.** 2014, Årsrapport SweTrau 20122013 per 131121 v2 [] Accessed February.

[B13] Advanced Trauma Life Support® Student Course Manual 2012 American College of Surgeons, Chicago, IL, USA

[B14] Pachter HL Hofstetter SR The current status of nonoperative management of adult blunt hepatic injuries Am J Surg 1995 169 442 454 10.1016/S0002-9610(99)80194-9 7694987

[B15] Pachter HL Guth AA Hofstetter SR Spencer FC Changing patterns in the management of splenic trauma: the impact of nonoperative management Ann Surg 1998 227 708 717 10.1097/00000658-199805000-00011 9605662 PMC1191351

[B16] Velmahos GC Toutouzas KG Radin R Chan L Demetriades D Nonoperative treatment of blunt injury to solid abdominal organs: a prospective study Arch Surg 2003 138 844 851 10.1001/archsurg.138.8.844 12912742

[B17] Franklin GA Casos SR Current advances in the surgical approach to abdominal trauma Injury 2006 37 1143 1156 10.1016/j.injury.2006.07.018 17092502

[B18] Smith J Armen S Cook CH Martin LC Blunt splenic injuries: have we watched long enough? J Trauma 2008 64 656 663 10.1097/TA.0b013e3181650fb4 18332805

[B19] Giannopoulos GA Katsoulis IE Tzanakis NE Patsaouras PA Digalakis MK Non-operative management of blunt abdominal trauma. Is it safe and feasible in a district general hospital? Scand J Trauma Resusc Emerg Med 2009 17 22 27 10.1186/1757-7241-17-22 19439091 PMC2689852

[B20] Parks NA Davis JW Forman D Lemaster D Observation for nonoperative management of blunt liver injuries: how long is long enough? J Trauma 2011 70 626 629 10.1097/TA.0b013e31820d1c69 21610353

[B21] Raza M Abbas Y Devi V Prasad KV Rizk KN Nair PP Non operative management of abdominal trauma – a 10 years review World J Emerg Surg 2013 8 14 10.1186/1749-7922-8-14 23561288 PMC3636075

[B22] Eppich WJ Zonfrillo MR Emergency department evaluation and management of blunt abdominal trauma in children Curr Opin Pediatr 2007 19 265 269 10.1097/MOP.0b013e328149af9e 17505184

[B23] Sartorelli KH Frumiento C Rogers FB Osler TM Nonoperative management of hepatic, splenic, and renal injuries in adults with multiple injuries J Trauma 2000 49 56 61 10.1097/00005373-200007000-00008 10912858

[B24] Lahiri R Bhattacharya S Pancreatic trauma Ann R Coll Surg Engl 2013 95 241 245 10.1308/003588413X13629960045913 23676806 PMC4132496

[B25] Degiannis E Glapa M Loukogeorgakis SP Smith MD Management of pancreatic trauma Injury 2008 39 21 29 10.1016/j.injury.2007.07.005 17996869

[B26] Hughes TM Elton C Hitos K Perez JV McDougall PA Intra-abdominal gastrointestinal tract injuries following blunt trauma: the experience of an Australian trauma centre Injury 2002 33 617 626 10.1016/S0020-1383(02)00068-2 12208066

[B27] Frick EJ Jr Pasquale MD Cipolle MD Small-bowel and mesentery injuries in blunt trauma J Trauma 1999 46 920 926 10.1097/00005373-199905000-00024 10338413

[B28] Fonseca AL Evans LV Gusberg RJ Open surgical simulation in residency training: a review of its status and a case for its incorporation J Surg Educ 2013 70 129 137 10.1016/j.jsurg.2012.08.007 23337682

[B29] Davies J Khatib M Bello F Open surgical simulation–a review J Surg Educ 2013 70 618 627 10.1016/j.jsurg.2013.04.007 24016373

